# Biodegradability of Novel Polylactide and Polycaprolactone Materials with Bacteriostatic Properties Due to Embedded Birch Tar in Different Environments

**DOI:** 10.3390/ijms221910228

**Published:** 2021-09-23

**Authors:** Agnieszka Richert, Agnieszka Kalwasińska, Maria Swiontek Brzezinska, Grażyna B. Dąbrowska

**Affiliations:** 1 Department of Genetics, Faculty of Biology and Veterinary Science, Nicolaus Copernicus University in Toruń, Lwowska 1, 87-100 Toruń, Poland; browsk@umk.pl; 2 Department of Environmental Microbiology, Faculty of Biology and Veterinary Science, Nicolaus Copernicus University in Toruń, Lwowska 1, 87-100 Toruń, Poland; kala@umk.pl (A.K.); swiontek@umk.pl (M.S.B.)

**Keywords:** biofilm, biodegradable films, birch tar, polycaprolactone, polylactide

## Abstract

The microbial biodegradation of new PLA and PCL materials containing birch tar (1–10% *v*/*v*) was investigated. Product of dry distillation of birch bark (*Betula pendula* Roth) was added to polymeric materials to obtain films with antimicrobial properties. The subject of the study was the course of enzymatic degradation of a biodegradable polymer with antibacterial properties. The results show that the type of the material, tar concentration, and the environment influenced the hydrolytic activity of potential biofilm degraders. In the presence of PCL films, the enzyme activities were higher (except for α-D-glucosidase) compared to PLA films. The highest concentration of birch tar (10% *v*/*v*) decreased the activity of hydrolases produced by microorganisms to the most significant extent; however, SEM analysis showed the presence of a biofilm even on plastics with the highest tar content. Based on the results of the biological oxygen demand (BOD), the new materials can be classified as biodegradable but, the biodegradation process was less efficient when compared to plastics without the addition of birch tar.

## 1. Introduction

There are many biodegradable polymer materials such as starch, cellulose, lignin, or aliphatic polyesters such as polylactide (PLA), poly (ε-caprolactone) (PCL) or poly (3-hydroxybutyric acid), obtained from renewable raw materials, which is of great importance in the age of fossil fuel depletion and the increasing problems with disposal of plastics. Among the materials listed above, the most important are polylactide (PLA) and polycaprolactone (PCL). They are relatively easily decomposed by microorganisms [1,2] and can be readily modified, which makes them more attractive than other materials. To protect the product against harmful microorganisms, various antimicrobial substances are introduced or applied to the polymeric materials, e.g., sorbic acid, benzoic acid, triclosan, grapefruit seed extract, lysozyme, and bacteriocins [3,4,5,6]. There are also many studies showing the use of volatile substances such as thymol, carvacrol [7,8,9,10], or nanoparticles, e.g., nanosilver [6,11,12], to obtain materials with bactericidal properties. Since 2013, more than 27,845 patents for antimicrobial polymers have been filed in the Google Patent Search database. In addition, antibacterial medical devices have attracted considerable attention in clinical trials [13]. Tar, a product of dry wood distillation, has been known for centuries and is used today in a variety of industrial sectors, mainly in cosmetics and pharmaceuticals [14,15]. This dark brown, thick liquid was used in the past to lubricate wooden gears, wheel hubs of carts and carriages, and to impregnate (“dressing”) buckskin leathers [16]. Recent studies suggest that tar was chewed in the Neolithic age to alleviate toothache [15,17]. Nowadays, purified and distilled tar is used in dermatology as an aseptic. It is used in the form of ointments and alcohol solutions in the treatment of skin diseases of humans and animals [18]. The suitability of birch tar oil as either a biocide, repellent against insects, weeds, and rodents, or in both, has also been documented [19,20,21]. Recently, attempts were made to use tar to protect plants against bacterial and fungal diseases [22,23,24,25] and to combat snails [26]. Biodegradation is defined as the breakdown of organic chemicals, including pollutants, into innocuous products such as CO_2_, CH_4_ and H_2_O by the action of microorganisms including archaea, bacteria and fungi. It depends on the activity of various enzymes. According to Zhuikov et al., (2021) [27], enzymatic degradation is found to significantly accelerate the degradation rate of PLA I PHB compared to non-enzymatic hydrolytic degradation. The first reported PLA-degrading enzyme was proteinase K from *Tritirachium album* [28,29]. Chemical modification of packaging materials is not always a preferred strategy in pollutant management and remediation as it may inhibit degradation by acting on the cells of microorganisms and their enzymes, particularly if the substance is of a strong biocidal nature and shows inhibitory activity towards biocatalysts. Environment conditions such as the content of organic matter, availability of elements, temperature, pH, and oxygen, influence the abundance of microorganisms and their metabolism, which determines the rate of biodegradation. Therefore, it is important to assess biodegradation of modified polymers thoroughly and especially under conditions stimulating those found in the natural environment.

The aim of this study was to demonstrate the biodegradability of new polylactide and polycaprolactone materials with embedded birch tar as biocidal substance in water and in soil environments. The subject of the study was the course of enzymatic degradation of a biodegradable polymer with antibacterial properties. We assume that PLA and PCL films, despite the presence of this antibacterial agent, will constitute a new type of biodegradable and cheap-to-produce ecological material that will be used in many industries.

## 2. Results

### 2.1. Description of the Produced Materials—Visual and Microscopic Analysis (SEM)

**Figure 1:**
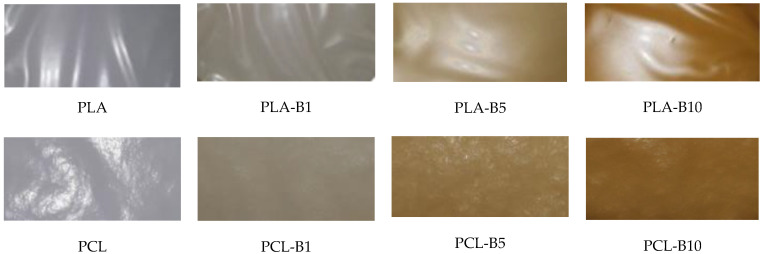
The appearance of the PLA and PCL films without and with birch tar.

A method of obtaining PLA and PCL films with the addition of birch tar has been developed. The increase in the tar content resulted in an increase in the color intensity of the foil (Figure 1). The films with 1% tar content were gray-brown in color, 5% content of this substance gave the film a light-brown color, and 10% of the addition of this substance made the films intensely brown.

**Figure 2:**
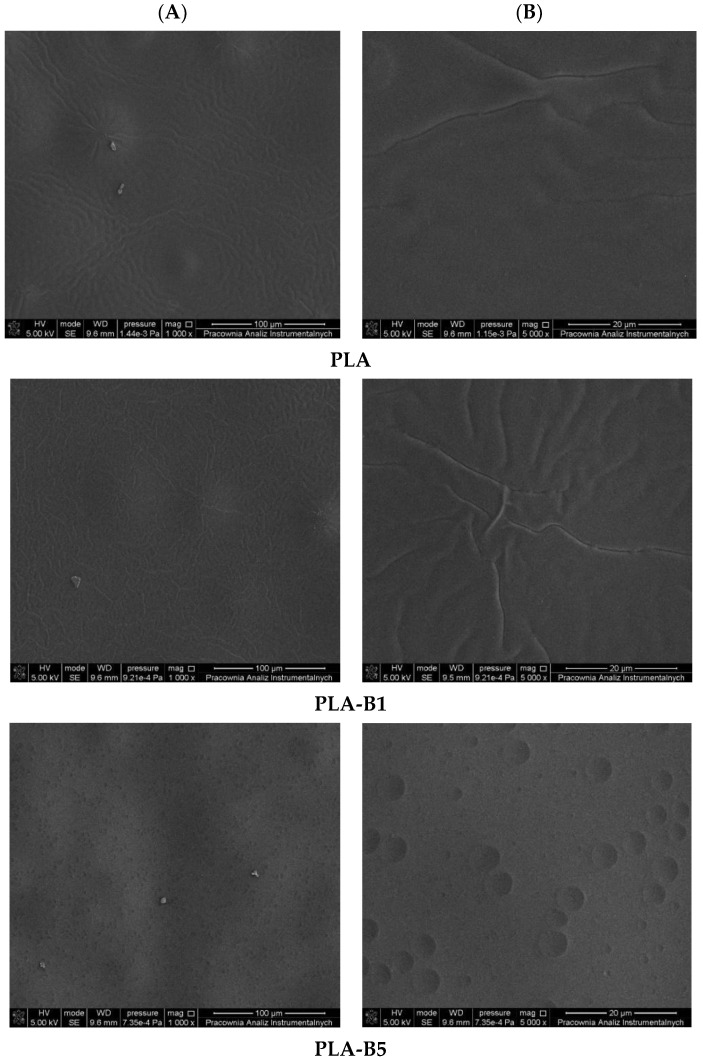

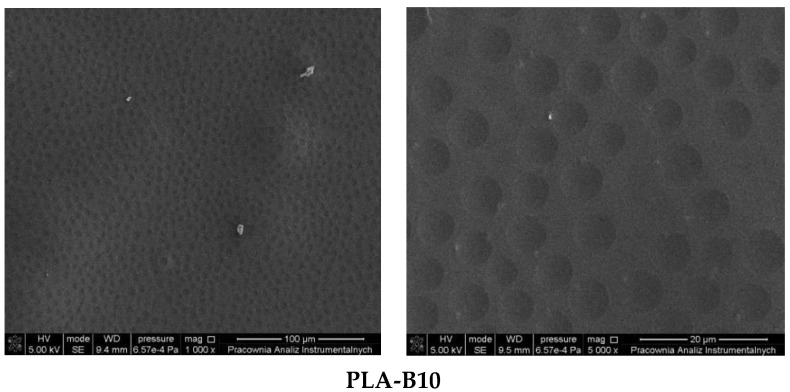
SEM analysis of surface of PLA and PLA with birch tar (PLA-B1–PLA-B10) materials, magnifications of 1000× (**A**) and 5000× (**B**).

**Figure 3:**
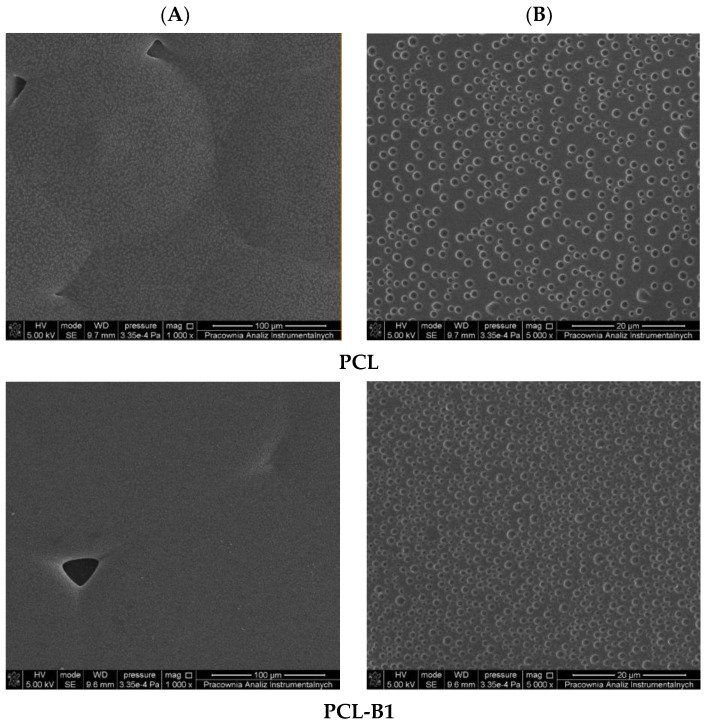

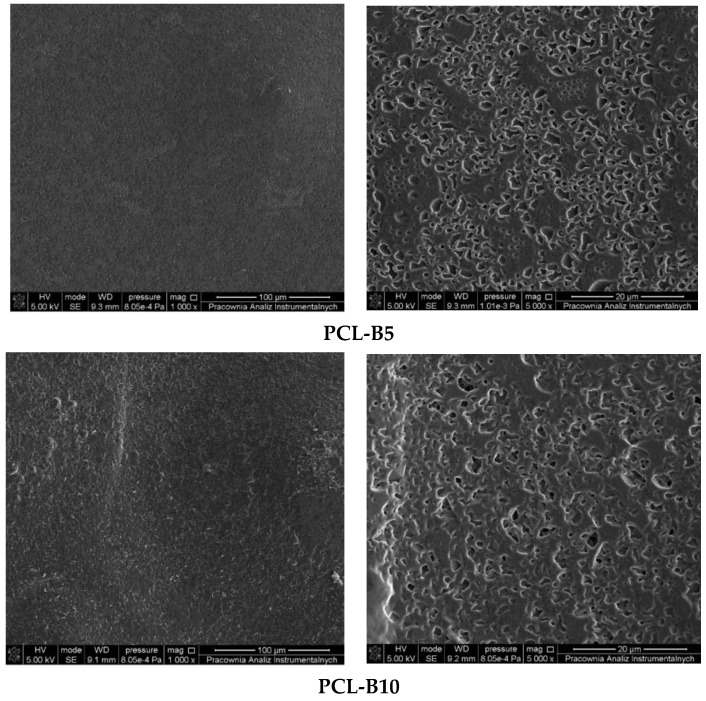
SEM analysis of surface of PCL and PCL with birch tar (PCL-B1–PCL-B10) materials, magnifications of 1000× (**A**) and 5000× (**B**).

Our research is aimed at the pursuit of new materials which have the ability to prevent bacteria from attaching themselves to polymeric surfaces. One of the possibilities is to obtain an antibacterial surface by introducing a biocidal agent into the polymer matrix. Figure 2 and Figure 3 show the surface morphology of the investigated surfaces.

In Figure 2 and Figure 3 SEM images of the studied PLA and PCL-based materials were shown. As can be observed, the material consisting of polylactide with an addition of plasticizer in the form of poly(ethylene glycol) is characterized by a rippled surface with irregularities indicating limited miscibility of the used polymers.

### 2.2. The Impact of Microorganisms from Various Environments on Materials Containing Birch Tar

Biological oxygen demand, biofilm, and enzymatic activity analyses were performed to demonstrate biodegradation changes.

#### 2.2.1. Biological Oxygen Demand

**Figure 4:**
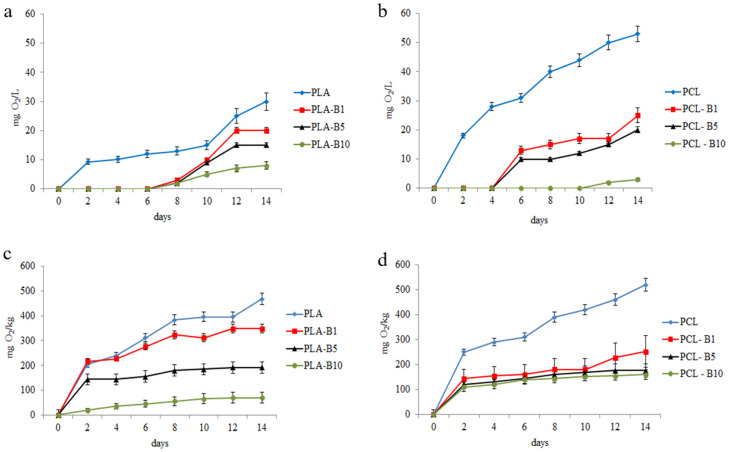
Biological oxygen demand (BOD7) in river water (**a**,**b**) and soil (**c**,**d**) in the presence of polymers; values are expressed as mean ± SD (*n* = 3).

To assess the biodegradability of PLA and PCL materials, biochemical oxygen demand was measured in water and soil in the presence of those materials (Figure 4).

It was shown that water microorganisms were characterized by higher metabolic activity (higher oxygen demand) in the presence of PCL, compared to PLA. The same tendency was observed for soil (Figure 4). BOD values obtained for soil were 10 times higher than in water. BODs from soil and water were also lower in the presence of polymers containing birch tar than in the presence of PCL and PLA without modifications. That means that birch tar reduces the rate of biodegradation. Water and soil microorganism are able to colonize materials which contain birch tar at the concentration of 1–10%.

#### 2.2.2. Biofilm Analysis Using SEM

**Figure 5:**
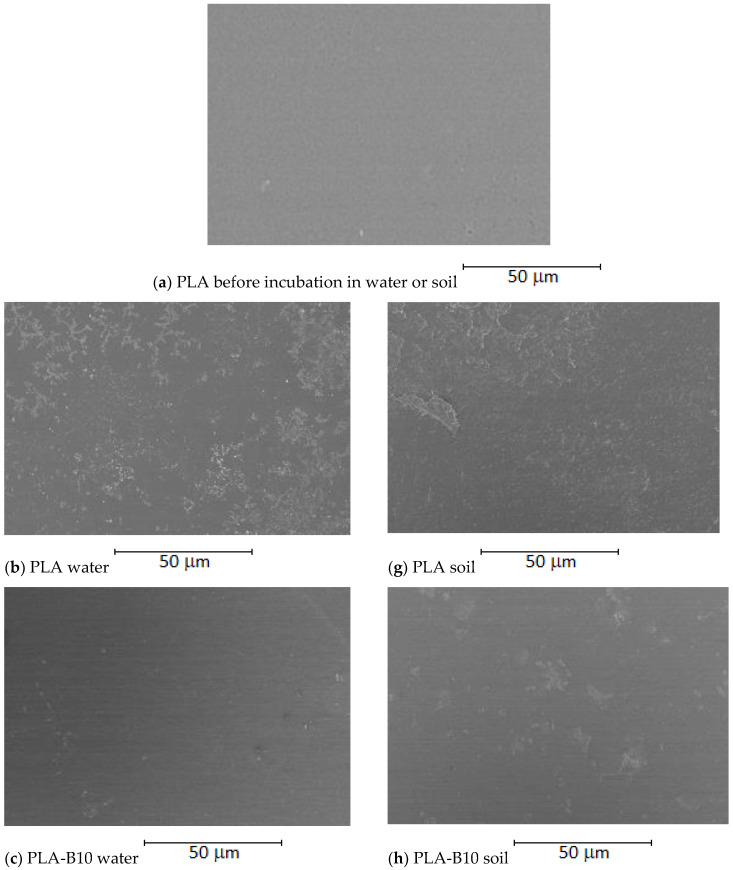

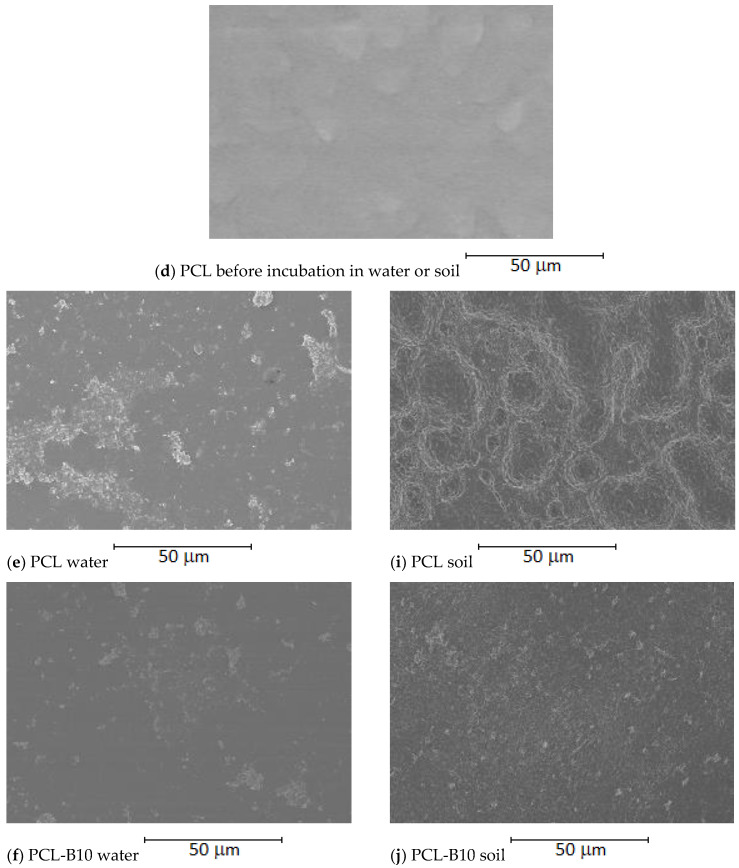
The biofilms formed on the surface of polymers and polymers with tar before biodegradation (**a**,**d**) and after biodegradation in water (**b**,**c**,**e**,**f**) and (**g**–**j**) in soil.

In order to analyze the surface morphology of the obtained polymeric films, scanning electron microscopy was applied. In Figure 5, SEM images of the studied PLA-based materials are shown.

#### 2.2.3. The Effect of Birch Tar Embedded into PLA and PCL on Bacterial Enzymatic Activity

**Figure 6:**
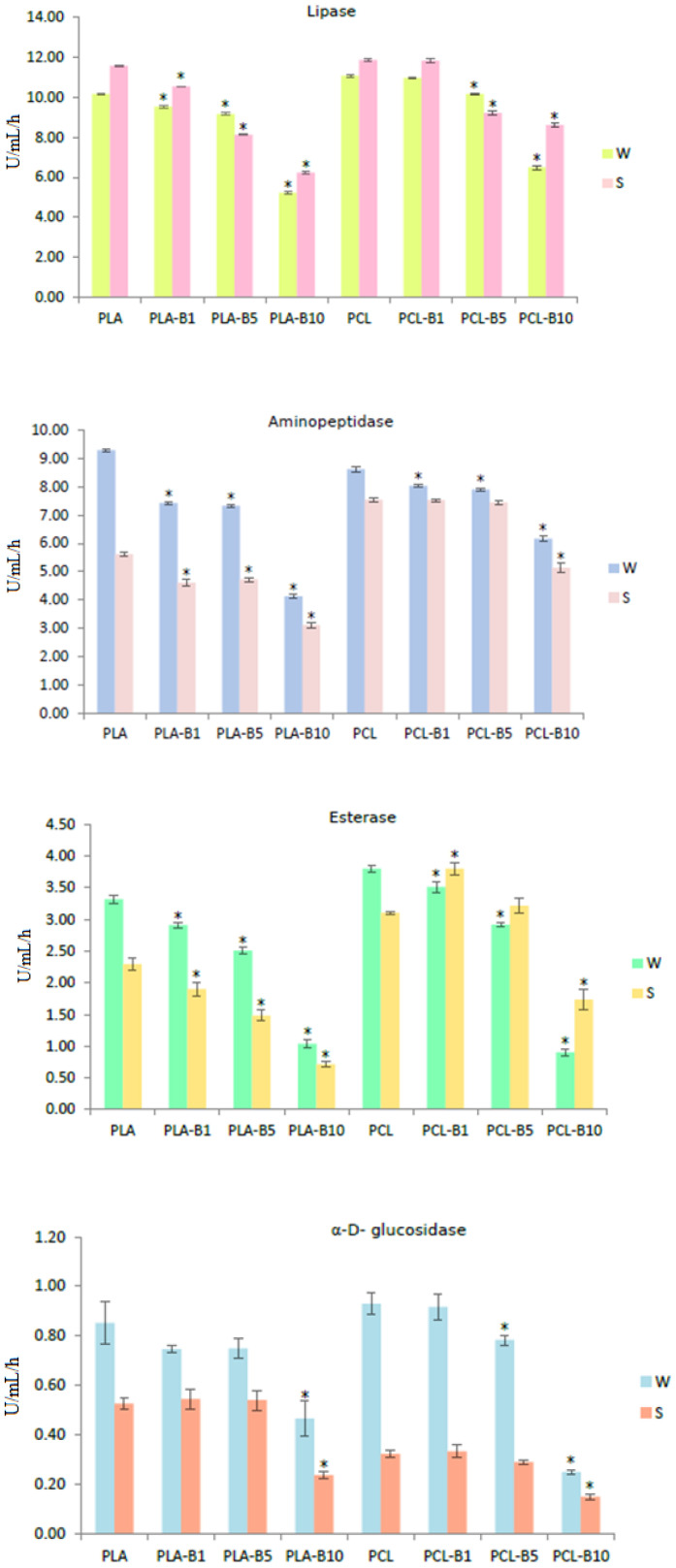

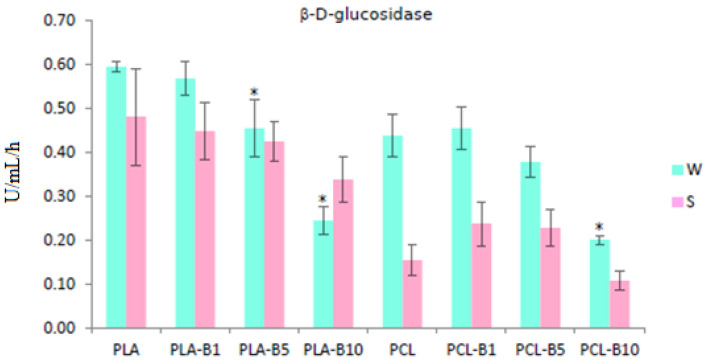
The enzymatic activity (lipase, aminopeptidase, esterase, α-D-glucosidase, and β-D-glucosidase) of biofilm-forming microorganisms on PLA, PCL, and films with birch tar, in river water and soil (* statistical differences).

The influence of tar embedded in PLA and PCL on the enzymatic activity of biofilms developed on the surface of the foils is presented in Figure 6.

The data shows that the activity of lipase, aminopeptidase, esterase, α-D-glucosidase, and β-D-glucosidase was affected by the birch tar concentration, and by the environment in which the films were deposited (Figure 6). All the enzymes showed a decrease in activity with an increase of the biocidal compound concentration. This decrease was linear except for β-D-glucosidase activity in the soil (Table 1). Mann–Whitney test for equal medians for enzyme activities in different materials and environments is presented in Table 2.

Most of the enzymes, except for lipase and aminopeptidase, showed higher activity in soil than in water. The activity of β-D-glucosidase was significantly higher in biofilm formed at PCL than PLA (Table 2). Among the five hydrolytic enzymes produced by microorganisms within the biofilm, the most active were lipase and aminopeptidase.

## 3. Discussion

Searching for new antibacterial agents that can be embedded into biodegradable materials is still in progress. The innovative polymers developed at the Department of Genetics of the Nicolaus Copernicus University are characterized by sound bactericidal properties, limiting the formation of biofilm. They are also biocompatible and show no genotoxicity and cytotoxicity.

Our previous studies have shown that birch tar incorporated into PLA and PCL films inhibited the growth of bacterial strains such as: *E. coli* and *S. aureus* [30,31]. Similarly, Shimizu et al., (2009) [32] showed that birch tar is one of the most effective natural compounds to kill *Legionella pneumophila*. Probably, the bioactive substances present in tar, such as phenolic compounds, are responsible for its biocidal properties.

New materials containing birch tar were characterized in our previous paper [31] (unpublished). We demonstrated the antibacterial properties of the new polymers as well as their chemical characteristics (water vapor permeability, FTIR, and AFM). In this paper we have focused on the surface analysis of the new PCL and PLA materials showing the structure of foil with the use of SEM as well as its susceptibility to microbial degradation.

Introduction of an antibacterial agent into the PLA-PEG system significantly influences the morphology of the obtained materials. The surface of PLA-based and PCL-based films with an addition of 1% wt of an antibacterial agent is covered by cracks more intensively than that of a film with a neat polymer matrix. Introduction of a higher content (5% and 10%) of birch tar into the PLA-PEG and PCL-PEG system results in the formation of holes, cavities and pores evenly distributed throughout the surface. Moreover, it should be emphasized that the number and size of cavities observed on the analyzed surfaces increased with the rise in the amount of the antibacterial agent added to the PLA-PEG and PCL-PEG systems. Interestingly, apart from the increase in the number of cavities, their size was also significantly increased. This can suggest that in the composition of birch bark certain volatile compounds are present which apparently evaporate during the formation of polymeric films. However, based on the extensive study, it was established that there is no single topographic pattern of materials characterized as favorable in terms of bacterial adhesion. Moreover, it was mentioned that, beside surface topography, the shape and size of bacteria substantially influence the interaction between the surface of polymeric material and the bacteria [1].

Heterotrophic microorganisms are present in every environment and their abundance and metabolic activity depends primarily on the amount of easily available organic matter. Bacterial densities in surface freshwaters such as rivers and lakes are usually lower (102–103 cfu/mL) compared to bottom sediments or soils (106–108 cfu/mL) [33]. Degradation of synthetic biopolymers is the outcome of the action of enzymes and acids secreted by microorganisms. Bacteria and fungi with the potential towards the degradation of high molecular weight biopolymers are not common in the environment as compared to microorganisms associated with low molecular weight biodegradable plastics [34]. Yet, their abundance is greater in soil than in water. Therefore, we have observed higher biological oxygen demand (BOD) in soil in the presence of tar-containing polymers than in water.

Previous research on biodegradation of PCL and PLA materials showed that several microbial hydrolytic enzymes are engaged in their degradation [35,36,37,38,39,40]. Our research showed that all the investigated enzymes secreted by microorganisms, both in soil and water, were inhibited in the presence of polymers containing birch tar, which is clearly related to the antibacterial action of tar.

Since information about the mechanism of antibacterial action of birch tar is not yet fully understood, we can only assume that it significantly affects the function of exoenzymes. It has been shown that compounds present in tar such as: phenolic compounds, cresols, allylphenol, guaiacol, 4-methyl- and 4-ethyl guaiacol, eugenol, isoeugenol, vanillin, and ethylvanillin have been identified in biomass pyrolysis of birch. The research by Czernik (2002) showed that phenols in the wood tar constitute up to 20–30% of its mass [41]. Rohn et al., (2002) showed that enzymes such as α-amylase can react with some phenolic compounds and inhibit their activity [42]. The decrease in the activity was accompanied by a reduction in the amount of free amino and thiol groups, as well as tryptophan residues, which resulted from the covalent attachment of the phenolic and related compounds to these reactive nucleophilic sites in the enzymes. Phenolics can also inactivate α-glucosidase and lipase through non-specific binding to enzymes [43]. Apart from phenolic compounds, some substances present in birch tar such as eugenol or vanillin have strong antimicrobial properties [44,45].

## 4. Materials and Methods

### 4.1. Materials

Polylactide (PLA) 2003D type (Ingeo Biopolymer 2003D, Nature Works LLC, Minnetonka, MN, USA) with a melt flow rate of 5–7 g 10 min^−1^ (2.16 kg; 190 °C) and a density of 1.24 g cm^−3^ was obtained. Poly(ε-caprolactone) (PCL), type CAPA 6800 (Solvay Caprolactones, Warrington, UK), MFI = 7.3 g/10 min (2.16 kg, 160 °C), Mn 69 kDa, d = 1.1 g/cm^3^. PLA and PCL were in the form of granules which were used to prepare the polymer solutions. Poly(ethylene glycol) (PEG) with Mw 1500 g mol^−1^ (Sigma-Aldrich Ltd., Poznań, Poland) was used as a plasticizer. Chloroform (Chempur, Piekary Sląskie, Poland) was used as a solvent. Birch tar (*Betula pendula*) (Poland) was used as an “antimicrobial” additive (agent).

### 4.2. Preparation of Films

The examined films were prepared using the solvent-casting method. For this purpose, polylactide pellets were dissolved in chloroform to obtain a 3% (*w*/*v*) polymer solution. Subsequently, 1 wt. %, 5 wt. % and 10 wt. % birch tar were added to the PLA or PCL solution. In total, 5 wt. % PEG was introduced into the solutions to prepare plasticized PLA- and PCL-based films and PLA and PCL-birch tar films. To obtain PLA-based materials, 50 mL of the prepared mixture was poured onto glass Petri dishes (14.5 cm in diameter) and left for 3 days to form a polymer film [31,46,47]. All symbols and the composition of the samples were presented in Table 1. The thickness of foils was measured with the use of electronic thickness gauge 0.001/0–12.7 mm Poland. They were in the range from 0.075 to 0.080 mm.

### 4.3. Biodegradation of Birch Tar-Containing PLA and PCL

Biodegradation of PLA and PCL with the embedded wood tar was determined based on the measurement of biological oxygen demand (BOD) with the OxiTop Control (WTW, Wrocław, Poland) according to the method described by Walczak et al., (2015) [48]. Biodegradation of films was studied in two environments, water and soil. The procedure for determining the biodegradation of birch tar containing PLA and PCL films in water was as follows. A 0.5 L bottle was filled with 430 mL of river water, a magnetic stirrer was placed inside the bottle, and five drops of nitrification inhibitor NT 600 (WTW, Wrocław, Poland) were added. Three fragments of the film (5 cm × 5 cm), and a rubber carrier containing CO_2_ absorbent (ca. 0.4 g NaOH) were then inserted into the bottle, and the OxiTop measuring heads were securely fitted. The biochemical oxygen demand was determined at 20 °C for 14 days. The control samples contained water without films. The samples were analyzed in three replicates. Biodegradation of the film in river water was expressed in mgO_2_/L of water after 14 days of incubation at the temperature of 20 °C. The biodegradation of PLA and PCL in soil was determined with a method modified by Platen and Wirtz (1999) [49]. One hundred grams of soil was placed in the 1 L jars. Three fragments of each film (5 cm × 5 cm) were then placed in the soil, and the carriers with absorber CO_2_ (0.4 g NaOH) were mounted to the lid of the jar. The samples were incubated at 20 °C for 14 days. The control sample was soil without films. Biodegradation of the film in soil was expressed in mgO_2_/kg soil after 14 days at the temperature of 20 °C. All the samples were analyzed in three replicates.

### 4.4. Scanning Electron Microscopy

The morphology of the films containing birch tar (before biodegradation in water and soil) was studied by means of the Quanta 3D FEG scanning electron microscope (SEM, FEI Company, Hillsboro, OR, USA). Photographs of the topography of the samples were taken using the SE detector in 5000-fold magnification. Before each of the analyses, the surfaces of the studied materials were sprayed with a Au layer.

Material structure analysis and biofilm identification on PLA and PCL with birch tar were performed using a scanning electron microscope (HITACHI SU 8010, Hitachi High-Technologies Co., Tokyo, Japan). The tests were carried out to determine the changes in morphology of the film surface after 7 days of incubation in the compost extract. In order to achieve the best quality photos, the film samples were previously sprayed with a Au/Pd alloy. Pictures taken at 1000×, 3000×, and 5000× magnification.

### 4.5. Determination of the Effect of Birch Tar on Enzymatic Activity of Biofilm

The enzymatic activity of biofilm was determined using 4-methylumbelliferyl (MUF) and 7-amido-4-methylcoumarin (AMC)-based substrates: 4-MUF-butyrate for lipase, 4-MUF-α-D-glucoside for α-glucosidase, 4-MUF-β-D-glucoside for β-glucosidase, and L-leucine 7-amido-4-methylcoumarin (AMC) for leucine-aminopeptidase [50]. All the substrates were purchased from Merck (Kenilworth, NJ, USA). Stock solutions of MUF and AMC substrates were prepared in methylcellosolve (ethyleneglycomonomethylether, EGME, Merck). The substrates were dissolved in 10 mL of methylcellosolve up to a concentration of 3 mM (L:1) and then stored at −20 °C. Before experimenting, stock solutions were thawed and diluted in sterile deionized water. Esterase activity was determined according to the modified Adam and Duncan method [51]. Titration plates (24-well plates) were used to determine the influence of tar on biofilm formation. Fragments of PLA and PCL (2 cm × 2 cm) with biofilm formed during incubation in water and soil were placed at the bottom of the well. Furthermore, 2 mL of broth medium were added and incubated for 24 h at 20 °C. After the incubation, 0.25 mL of an appropriate substrate was added to each well (final concentration of 50 µmol/L). The enzymatic reaction was carried out at 40 °C for one hour. MUF or AMC concentration was determined using the Hitachi F2500 spectrofluorometer (Tokyo, Japan). One unit of enzyme activity (U/mL/h) was defined as 1 nmol MUF or AMC released per ml per h [52]. To determine the esterase activity, fragments of the films (2 cm × 2 cm) were placed in titrate plate with broth medium at 20 °C for 24 h. After adding 50 µL of fluorescein diacetate (1 mg/mL), the plates were incubated at 40 °C for one hour. The concentration of fluorescein was measured using a HITACHI F2500 spectrofluorometer at a 480 nm excitation wavelength and 505 nm emission wavelength. The unit of hydrolase activity was expressed as µg of fluorescein per ml per hour (U/mL/h). All the enzymatic analyses were performed in triplicate.

### 4.6. Statistical Analyses

All the analyses were performer using Past v. 3.08 (Hammer et al., 2015). The normality of distribution and homogeneity of variances were checked using Shapiro–Wilks and Levene’s tests, respectively. To determine the effect of environment (water and soil) and type of the material (PLA and PCL) on respiration, biofilm, and enzyme activity, two-way ANOVA was applied. The least squares regression was performed to predict the behavior of variables mentioned above in relations to the birch tar concentration.

## 5. Conclusions

Tar-containing materials are a promising alternative to plastics containing substances with biocidal properties of chemical origin. The subject of the study was the course of enzymatic degradation of a biodegradable polymer with antibacterial properties. New plastics based on PLA with the addition of tar were produced and their structure was analyzed with the use of SEM. The application of the BOD technique has shown that microorganisms from the aquatic environment and soil are able to grow on tar plastics and to biodegrade them. Tar contained in the films limited the growth of microorganisms, especially those coming from the soil. Microscopic observations of new materials showed the presence of biofilm, which proves the ability of microorganisms to biodegrade the foil with tar. Although a decrease in the activity of hydrolytic enzymes was noted in the presence of films with embedded tar, it was not sufficiently significant to prevent biodegradation, as indicated by measurements of respiration. Additionally, it was shown that biofilm can be formed at the surface of the polymers that gives a future chance for retrieving specific strains of bacteria having the ability to easily decompose such materials that may significantly accelerate their degradation.

## 6. Patents

Richert, A., Dąbrowska, G.B., Dąbrowski, H.P., 2020. Bactericidal polylactide film and the method of its preparation. Patent Application P.433979 (in Polish).

## Figures and Tables

**Table 1:** Least square linear regression analyses of tar concentration and enzyme activities.

Film and Environment	Parameter	Lipase	Amino-Peptidase	Esterase	α-D-Gucosidase	β-D-Glucosidase
PLA water	Slope a:	−0.46306	−0.44032	−0.21339	−0.03393	−0.03484
	Intercept b:	10.365	8.8021	3.2944	0.83987	0.60352
	r^2^:	0.89	0.879	0.96	0.78	0.93
	*p*	<0.0001	<0.0001	<0.0001	<0.0001	<0.0001
PCL water	Slope a:	−0.45022	−0.22081	−0.28022	−0.06753	−0.02468
	Intercept b:	11.467	8.5607	3.9009	0.99011	0.46538
	r^2^:	0.91	0.90	0.95	0.91	0.85
	*p*	<0.0001	<0.0001	<0.0001	<0.0001	<0.0001
PLA soil	Slope a:	−0.52075	−0.20855	−0.14661	−0.02737	−0.01317
	Intercept b:	11.191	5.345	2.1848	0.57113	0.47435
	r^2^:	0.97	0.83	0.96	0.65	0.43
	*p*	<0.0001	<0.0001	<0.0001	<0.01	<0.05
	Slope a:	−0.3543	−0.23425	−0.16446	−0.0178	−0.00737
PCL soil	Intercept b:	11.784	7.8487	3.6212	0.34535	0.2103
	r^2^:	0.89	0.80	0.72	0.88	0.22
	*p*	<0.0001	<0.0001	<0.0001	<0.0001	0.125

**Table 2:** Mann-Whitney test for equal medians for enzyme activities in different materials and environments.

	Enzyme	Mann-Whitney U	*p*
PLA vs. PCL	Lipase	178.5	<0.05
	Amino	122.0	<0.01
	Esterase	135.5	<0.01
	α-D-Glucosidase	236.0	ns
	β-D-Glucosidase	105.0	<0.001
Water vs. Soil	Lipase	237.0	ns
	Amino	139.0	<0.01
	Esterase	231.5	ns
	α-D-Glucosidase	78.0	<0.0001
	β-D-Glucosidase	158.5	<0.01

ns—not significant.

## Data Availability

All data is contained within the manuscript.
